# Correction: Measuring trade costs and analyzing the determinants of trade growth between Cambodia and major trading partners: 1993–2019

**DOI:** 10.1371/journal.pone.0348702

**Published:** 2026-04-30

**Authors:** Borin Keo, Bin Li, Waqas Younis

The Data Availability statement for this article is incorrect. The correct statement is: All relevant data used in this study are freely available from public sources, including the Direction of Trade Statistics (DOTS) database of the International Monetary Fund (IMF), the OECD Structural Analysis (STAN) databases, the UNCTAD database, the International Trade and Production Database for Estimation (ITPD-E-R02), and the IMF World Economic Outlook (WEO) database, as described in the original publication.

In addition, the fully compiled dataset supporting the results of this study complies with relevant academic data-sharing regulations and is publicly available in the Zenodo repository without access restrictions at https://zenodo.org/records/15253951 (DOI: 10.5281/zenodo.15253951). The dataset will be permanently preserved to support research reproducibility and independent verification.
