# Supplementary material for: Measuring trade costs and analyzing the determinants of trade growth between Cambodia and major trading partners: 1993–2019
Source: PLoS One. 2025 Jan 24;20(1):e0311754. doi: 10.1371/journal.pone.0311754 (PMC11760036; doi:10.1371/journal.pone.0311754)
Supplement: S1 Appendix — (DOCX) [file pone.0311754.s001.docx]

**S1 Appendix: Details of the data descriptions**

The primary source of the bilateral export data in free on board (FOB) is derived from the IMF's Direction of Trade Statistics (DOTS) because the IMF's DOTS database is the most widely used database for the aggregate bilateral trade data for international trade research [1,2]. However, the data of certain trading partners in specific years are not available from the IMF's DOTS database. Therefore, where possible, the missing data are first supplemented using the data from the Organization for Economic Co-operation and Development (OECD) STAN Database. If the data on exports are not available from the OECD STAN database, they are complemented by the United Nations Conference on Trade and Development (UNCTAD) database.

The IMF's DOTS database does not include information on the exports (FOB) from the Taiwan Province of China to Cambodia from 1993 to 2019, the exports from the Republic of Korea to Cambodia, and from Cambodia to the Republic of Korea from 1995 to 1996, as well as the export data from Norway to Cambodia in 2000. Therefore, we use the data derived from the OECD STAN database to fill in the missing. The exports from Cambodia to New Zealand in 2009 and 2010, the export data from the Russian Federation to Cambodia in 1995, and the exports from Cambodia to the Russian Federation in 1995 and 1997 are complemented by the data from the UNCTAD. The IMF's DOTS database does not contain the exports (FOB) from Cambodia to the Republic of Korea before 1997. Similarly, the export data in FOB that Cambodia reports are not available from the OECD STAN database before 2000, so the import data in cost, insurance, and freight (CIF) reported by the Republic of Korea are used instead. As equation (8) suggests, the export data in FOB are recommended to avoid extra costs. However, a few missing data supplemented using the data from trading partners do not influence our results. When using this approach to fill in the missing data for relevant years, the errors are minimal.

The domestic trade data are obtained at the latest update (second edition) from the International Trade and Production Database for Estimation Release 2 (ITPD-E-R02), published in July 2022. The ITPD-E-R02 database covers the data for 265 countries or regions and 170 industries in the four sectors, including agriculture, mining & energy, manufacturing, and services from 1986 to 2019 (refer to Borchert et al. [3] for the details on how the data are constructed). It is important to emphasize that the scope of this study is limited to trade in the goods sector and total trade costs. Due to the limited data availability on domestic trade in other sectors for Cambodia and other developing countries, this study does not examine disaggregated trade costs by industries. To obtain the overall domestic trade data in the goods sector, we aggregate the three industries, including agriculture, mining & energy, and manufacturing, which are available from the ITPD-E-R02 database. The domestic trade data obtained from the ITPD-E-R02 are not available for Hong Kong (China) from 1993 to 1996 and 2019, Cambodia in 2019, and India in 2019, so these missing data are filled in using the interpolation method.

The GDP data are also needed to decompose the growth of the Cambodian trade using equation (16). All the GDP data are taken from the IMF's World Economic Outlook (WEO) database, released in October 2022. To capture the price indices in what Anderson and van Wincoop [4] refer to as "multilateral resistance", trade volumes and incomes are estimated using the nominal value. All the data are measured in thousands of current U.S. dollars for the corresponding year. It is important to note that the databases from the IMF's DOTS and the ITPD-E-R02 report the data on Belgium-Luxembourg as a group from 1993–1996 and 1993–1998, respectively. Therefore, to make it consistent with all the variables required in the analysis, the data for Belgium are calculated as Belgium and Luxembourg from 1993–1998.

**References**

1. Huot N, Kakinaka M. Trade Structure and Trade Flows in Cambodia: A Gravity Model. ASEAN Economic Bulletin. 2007;24: 305–319.

2. Novy D. Gravity redux: measuring international trade costs with panel data. Economic Inquiry. 2013;51: 101–121. doi:10.1111/j.1465-7295.2011.00439.x

3. Borchert I, Larch M, Shikher S, Yotov YV. The International Trade and Production Database for Estimation - Release 2 (ITPD-E-R02). Washington, DC: U.S. INTERNATIONAL TRADE COMMISSION; 2022 Jul. Report No.: 2022–07–A. Available: https://www.usitc.gov/publications/332/working_papers/itpd_e_r02_usitc_wp.pdf

4. Anderson JE, van Wincoop E. Gravity with Gravitas: A Solution to the Border Puzzle. The American Economic Review. 2003;93: 170–192. doi:10.1257/000282803321455214
